# Disseminated *Mycobacterium tilburgii* infection complicated by pulmonary non-tuberculosis mycobacteriosis in a patient with acquired immunodeficiency syndrome: A case report and literature review

**DOI:** 10.1097/MD.0000000000047989

**Published:** 2026-03-13

**Authors:** Yuki Sakai, Miki Miyazawa, Akifumi Hisada, Yukari Tanabe, Satoshi Arakawa, Tsukasa Nozaki, Hidetaka Yanagi, Kentaro Wakamatsu, Akiko Takaki, Satoshi Mitarai, Kazuo Umezawa, Satomi Asai

**Affiliations:** a Department of Laboratory Technology, Tokai University Hospital, Isehara City, Kanagawa, Japan; b Department of Internal Medicine, Tokai University School of Medicine, Isehara City, Kanagawa, Japan; c Department of Laboratory Medicine, Tokai University School of Medicine, Isehara City, Kanagawa, Japan; d Department of Respiratory Medicine, National Hospital Organization Omuta National Hospital, Fukuoka City, Japan; e Department of Mycobacterium Reference and Research, The Research Institute of Tuberculosis, Japan Anti-Tuberculosis Association, Kiyose-shi, Tokyo, Japan; f Department of Emergency and Critical Care, Tokai University School of Medicine, Isehara City, Kanagawa, Japan.

**Keywords:** bloodstream infection, case report, complicated infections, *Mycobacterium tilburgii*, non-tuberculosis

## Abstract

**Rationale:**

*Mycobacterium tilburgii* is the causative agent of disseminated non-tuberculosis mycobacterial infections in individuals who are immunocompromised, including those with human immunodeficiency virus infection. Owing to its non-culturable nature, identification of *M. tilburgii* relies solely on genetic analysis, making reports of *M. tilburgii* infections rare. We report a case of disseminated *M. tilburgii* infection complicated by a mixed pulmonary non-tuberculosis mycobacterial infection in a patient with acquired immunodeficiency syndrome.

**Patient concerns:**

A male patient in his 40s, diagnosed with human immunodeficiency virus, was admitted with suspected disseminated non-tuberculosis mycobacterial infection based on initial laboratory findings and mycobacterial testing. *Mycobacterium intracellulare* and *Mycobacterium kansasii* were detected in the respiratory specimens. Acid-fast staining of the blood and bone marrow samples was positive; however, no bacterial growth was observed in the cultures. Genetic analysis of the blood and bone marrow samples revealed the presence of *M. tilburgii*.

**Diagnoses:**

The patient was diagnosed with disseminated *M. tilburgii* infection, accompanied by a mixed pulmonary non-tuberculosis mycobacterial infection. *Enterococcus faecium*, *Candida parapsilosis*, and *Candida glabrata* were also detected in blood cultures.

**Outcomes:**

Despite ongoing treatment with antibiotics and antifungals, the patient died of septic shock.

**Lessons:**

*Mycobacterium tilburgii* is non-culturable; therefore, when acid-fast bacilli are detected in smear microscopy without subsequent culture growth, clinicians should consider the possibility of *M. tilburgii* infection and conduct thorough investigations.

## 1. Introduction

*Mycobacterium tilburgii* was reported in the 1990s as a non-culturable mycobacterium that can only be detected via genetic analysis.^[1,2]^ It causes disseminated non-tuberculosis mycobacterial (DNTM) infections in the lungs, lymph nodes, and gastrointestinal tract. Individuals who are immunocompromised, including patients with human immunodeficiency virus (HIV) infection or Mendelian susceptibility to mycobacterial disease, are particularly vulnerable to DNTM infections.^[3]^ Owing to its non-culturable nature, reports of *M. tilburgii* infections are rare. We report a case of disseminated *M. tilburgii* infection complicated by a mixed pulmonary non-tuberculosis mycobacterial (NTM) infection in a patient with acquired immunodeficiency syndrome (AIDS).

## 2. Case presentation

A Japanese male patient in his 40s presented with worsening epigastric pain to the emergency department of Tokai University Hospital. He was diagnosed with HIV infection 1 year before the emergency room visit and had undergone anti-retroviral treatment with tenofovir alafenamide/emtricitabine and dolutegravir; however, he had discontinued follow-up visits after 1 month of initial treatment, and the HIV infection was speculated to be poorly controlled. On admission, his vital signs and demographics were as follows: height, 165 cm; weight, 54 kg; body temperature, 38.1 °C; blood pressure, 143/96 mm Hg; heart rate, 66 beats/min; respiratory rate, 20 breaths/min; and oxygen saturation, 91% on room air. Laboratory test results revealed anemia, thrombocytopenia, and elevated inflammatory marker levels (Table 1). Flow cytometric analysis of the peripheral blood revealed a low CD4 count (38 cells/µL) and decreased CD4/CD8 ratio, consistent with AIDS. The patient was positive for HIV-1/2 antibody and *Aspergillus* antigen but had negative results in an interferon-gamma release assay (enzyme-linked immunospot [ELISPOT]) and was negative for *Mycobacterium avium* complex (MAC) antibodies. Furthermore, β-d-glucan was within the normal range, whereas the procalcitonin level was mildly elevated.

**Table 1:** Laboratory data on admission.

Hematological laboratory findings		Biochemical laboratory findings		Infectious disease laboratory findings		Blood cell surface marker test findings	
WBC	7.3 × 10^3^ μL	Total protein	6.0 g/dL	Procalcitonin	0.442 ng/mL	CD4/CD8 ratio	0.15
RBC	2.12 × 10^6^ μL	Albumin	1.0 g/dL	TP antibody	(−)	CD4	9.8%
Hemoglobin	3.4 g/dL	Creatine kinase	35 U/L	HBs antigens	(−)	CD8	67.1%
Hematocrit	13.5%	Aspartate aminotransferase	11 U/L	HBc antibodies	(−)	CD4 count	38 cells/µL
MCV	63.7 fL	Alanine aminotransferase	8 U/L	HCV antibodies	(−)	CD8 count	263 cells/µL
MCH	16.0 pg	Lactate dehydrogenase	170 U/L	HIV-1/2 antibodies	(+)		
MCHC	25.2%	Alkaline phosphatase	84 U/L	HIV-1 RNA qPCR	60,000 copy/mL		
Platelet	3.2 × 10^4^ μL	Blood urea nitrogen	12 mg/dL	β-D-Glucan	3.1 pg/mL		
Blood imaging findings		Na	135 mmol/L	IGRA (ELISPOT)	(−)		
Segment	90.4%	K	3.5 mmol/L	MAC antibodies	(−)		
Lymphocyte	5.8%	Cl	106 mmol/L	*Aspergillus* antigens	(+)		
Monocyte	3.8%	C-reactive protein	4.99 mg/dL				

HBc-Ab = hepatitis B core, HBs = hepatitis B surface, HIV = human immunodeficiency virus, IGRA = interferon-gamma release assay, MAC = *Mycobacterium avium* complex, MCH = mean corpuscular hemoglobin, MCHC = mean corpuscular hemoglobin concentration, MCV = mean corpuscular volume, qPCR = quantitative polymerase chain reaction, RBC = red blood cell, RNA = ribonucleic acid, TP = Treponema pallidum, WBC = white blood cell.

Sputum and gastric fluid samples were treated with *N*-acetyl-l-cysteine-sodium hydroxide and smeared onto glass slides. Blood and bone marrow aspirates were vigorously mixed with distilled water and centrifuged at 3000 × *g* and 4 °C for 20 min. The resulting pellets were resuspended and smeared onto slides. Bone marrow samples were homogenized and smeared onto glass slides. All prepared specimens were stained using the Ziehl–Neelsen method.^[4]^

**Figure 1.**
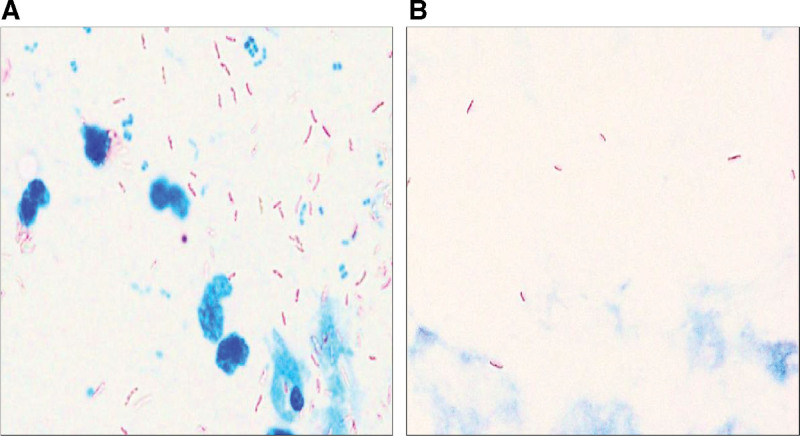
Ziehl–Neelsen staining of specimens. Over 10 acid-fast bacilli per field of view were detected in the sputum specimen, some of which exhibited a crossbanding-like striped morphology (A). Several elongated acid-fast bacilli per field of view were detected in the bone marrow and bone marrow aspirate samples (B).

On hospital day 1, >10 acid-fast bacilli per field of view (3+) were detected in the sputum specimen, exhibiting a crossbanding-like striped morphology (Fig. 1A). Additionally, 1 to 9 acid-fast bacilli per 100 fields of view (1+) with similar crossbanding were detected in the gastric fluid sample. On the second day of illness, 1 to 2 elongated acid-fast bacilli per 300 fields of view (+/−) were detected in the blood sample, whereas >10 elongated acid-fast bacilli per field of view (3+) were detected in the bone marrow and bone marrow aspirate samples (Fig. 1B). The bacilli in the bone marrow specimens did not exhibit the same crossbanding as those in the sputum specimens, indicating staining differences between these sources.

Sputum and gastric fluid samples were processed using the *N*-acetyl-l-cysteine-sodium hydroxide method^[5]^ and cultured using the BD BACTEC mycobacterium growth indicator tube (MGIT) 960, a fully automated mycobacterial culture system, with MGITs (Becton, Dickinson and Co., Franklin, NJ, USA; Table 2). Blood and bone marrow aspirates were vigorously mixed with distilled water and centrifuged at 3000 × *g* and 25 °C for 20 min. The resulting pellets were cultured in MGIT with the BACTEC system. Additionally, the bone marrow samples were homogenized and cultured in MGIT with the BACTEC system.

**Table 2:** Mycobacterial test results.

	Specimen	Smear positivity	LAMP method	PCR method	Cultivation results using mass spectrometry	Genetic analysis (NGS)	
						Gene target	Result/organism identified
Day 1	Sputum	3+	Negative	*Mycobacterium intracellulare*	*Mycobacterium intracellulare* and *Mycobacterium kansasii*	Not implemented	
	Gastric juice	1+	Negative	*Mycobacterium intracellulare*	*Mycobacterium intracellulare* and *Mycobacterium kansasii*	Not implemented	
	Cerebrospinal fluid	−	Negative	Negative	Negative	Not implemented	
	Blood*	±	Negative	Negative	Negative	*hsp65*	*Mycobacterium simiae* (homology, 96.01%)
						16S rRNA**	*Mycobacterium tilburgii* (homology, 100.0%)
Day 2	Sputum	2+	Not implemented	Not implemented	*Mycobacterium intracellulare* and *Mycobacterium kansasii*	Not implemented	
	Bone marrow aspirate*	3+	Negative	Negative	Negative	*hsp65*	*Mycobacterium simiae* (homology, 96.509%)
						16S rRNA**	*Mycobacterium tilburgii* (homology, 100.0%)
	Bone marrow	3+	Negative	Negative	Negative	Analysis not performed owing to insufficient number of bacteria.	
Day 5	Sputum	3+	Negative	*Mycobacterium intracellulare*	*Mycobacterium intracellulare* and *Mycobacterium kansasii*	Not implemented	
Day 30	Sputum*	Not implemented	Not implemented	Not implemented	*Mycobacterium intracellulare*	*hsp65*	*Mycobacterium simiae* (homology, 96.01%) *Mycobacterium intracellulare* (homology, 99.002%)
						16SrRNA**	Mycobacterium tilburgii (homology 100.0%)

LAMP = loop-mediated isothermal amplification, NGS = next-generation sequencing, PCR = polymerase chain reaction.

* NGS analysis was initiated on hospital day 30, using blood and bone marrow aspirates collected on day 1 and sputum collected on day 30.

** Results of the additional 16S rRNA gene analysis for the strain that showed > 96% sequence similarity with *M. simiae* in the *hsp65* gene analysis, conducted using the Deeplex Myc-TB assay.

The loop-mediated isothermal amplification method (Loopamp EXIA, Loopamp Mycobacterium Detection Kit, Eiken Chemical Co., Ltd., Tokyo, Japan) was used to identify *Mycobacterium tuberculosis*. All specimens tested negative for *M. tuberculosis*. MAC (*Mycobacterium intracellulare*) was detected in the sputum and gastric fluid samples using real-time polymerase chain reaction (PCR; Cobas TaqMan48, TaqMan MAI, Roche, Basel, Switzerland). Specifically, MAC was undetected in the blood, bone marrow aspirate, and bone marrow samples.

In the culture tests, the sputum sample from day 1 yielded positive results after 9 days of incubation, whereas the gastric fluid sample yielded positive results after 7 days of incubation. The MGIT culture was subcultured on 2% Ogawa medium (Kyokuto Pharmaceutical Industrial Co., Ltd., Tokyo, Japan) and Middlebrook 7H11 agar (Becton, Dickinson and Co.) and incubated at 37 °C under aerobic conditions for 3 weeks. Both white and yellow colonies were observed on solid media. The yellow and white colonies were identified as *Mycobacterium kansasii* (score value: 2.02) and *Mycobacterium chimaera intracellulare* group (score value: 2.20), respectively, using MALDI Biotyper (Bruker Corp., Billerica, MA, USA).

Acid-fast bacillus testing of sputum samples collected on days 2 and 5 revealed the presence of *M. intracellulare* and *M. kansasii*. However, no growth was observed in the blood samples collected on day 1 or in the bone marrow and bone marrow aspirates collected on day 2 after 14 days of incubation.

The presence of mycobacteria that are unable to grow in the MGIT system was suspected. Therefore, to investigate the possibility of *Mycobacterium haemophilum* infection, specimens were cultured using 5% sheep blood agar (Kyokuto Pharmaceutical Industrial Co.), 5% chocolate agar (Kyokuto Pharmaceutical Industrial Co.), 2% Ogawa medium, and Strepto-hemosupplement (Eiken Chemical Co., Ltd., Tokyo, Japan). Subsequently, 650 µL of each medium was added to MGITs, and cultures were incubated under aerobic conditions at 30 and 37 °C. Notably, no growth of acid-fast bacilli was observed in any of the cultures after 3 months.

Genetic analyses were initiated on hospital day 30, using blood and bone marrow aspirates collected on hospital day 1 and sputum collected on hospital day 30. Testing was performed using targeted next-generation sequencing (NGS; Deeplex Myc-TB, GenoScreen, Lille, France) and the iSeq 100 System (Illumina, Inc., San Diego, CA, USA). Mycobacteria with > 96% homology to *Mycobacterium simiae* were detected by analyzing the *hsp65* gene. Conversely, those with high homology to *M. simiae* in the *hsp65* gene showed 100% homology to *M. tilburgii* in the 16S ribosomal RNA (rRNA) gene. No pathogens were detected in the bone marrow owing to insufficient bacterial load. The housekeeping gene (16S rRNA) matched that of *M. tilburgii*, and no growth was observed in cultures under multiple conditions, confirming the organism as *M. tilburgii*.

*Mycobacterium intracellulare* and *M. kansasii* strains isolated from the sputum on day 1 were subjected to antimicrobial susceptibility testing using Broth MIC NTM (Kyokuto Pharmaceutical Industrial Co.; Table 3). However, testing for *M. tilburgii* was not feasible owing to the absence of growth in the culture.

**Table 3:** Antimicrobial susceptibility testing of *Mycobacterium intracellulare* and *Mycobacterium kansasii* from the sputum.*

Antimicrobial agent		*Mycobacterium intracellulare*	*Mycobacterium kansasii*
		MIC (μg/mL)	MIC (μg/mL)
Streptomycin	(STR)	2	16
Ethambutol	(EMB)	4	4
Kanamycin	(KM)	2	64
Rifampicin	(RIF)	0.25	0.125
Rifabutin	(RBT)	0.125	0.03
Levofloxacin	(LVX)	1	0.5
Clarithromycin	(CLR)	0.06	0.125
Ethionamide	(ETH)	1	1
Amikacin	(AMK)	2	8

MIC = minimum inhibitory concentration.

* Because *M. tilburgii* is non-culturable, antimicrobial susceptibility testing could not be performed.

**Figure 2.**
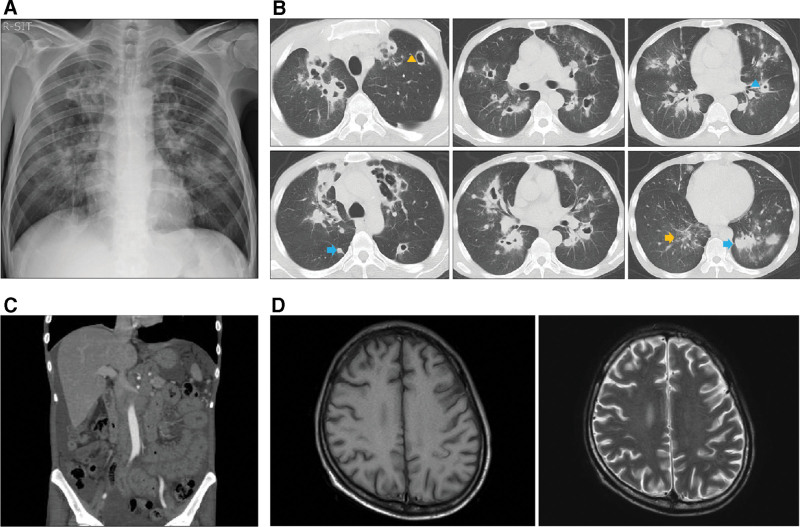
Imaging findings on admission. Chest radiograph revealed cavitary lesions in both lungs (A). Chest computed tomography confirmed multiple cavitary lesions in both lungs, ranging from thin-walled to thick-walled lesions. Nodular (blue arrows) and granular (yellow arrows) opacities with relatively well-defined borders and high contrast were also observed. Many nodules and granules showed a centrilobular distribution, whereas some nodules were suspected to have a random distribution. Cavitary lesions were also observed (yellow arrowhead) (B). Abdominal imaging revealed intra-abdominal lymphadenopathy and edematous thickening of the small intestinal wall (C). White matter lesions were observed in the left parietal lobe (D).

The patient CD4 count was low (38 cells/µL) during the initial examination. Imaging studies, including chest radiography (Fig. 2A), computed tomography (Fig. 2B), and magnetic resonance imaging (Figure 2C, D), revealed pulmonary, abdominal, and cerebral abnormalities, including cavitary lesions in the lungs, intra-abdominal lymphadenopathy, and a white matter lesion in the left parietal lobe. Based on the imaging results for AIDS, DNTM infection or miliary tuberculosis was suspected. On day 4, cryptococcal meningitis was excluded as a differential diagnosis, and anti-retroviral therapy with tenofovir disoproxil fumarate/emtricitabine and dolutegravir was initiated.

*Mycobacterium intracellulare* was detected in the sputum and gastric fluid; therefore, a disseminated MAC infection was suspected. Consequently, the patient was treated with clarithromycin (800 mg/day), ethambutol (750 mg/day), and rifabutin (300 mg/day) beginning on day 6. Despite a reduction in HIV viral load following continued anti-retroviral therapy, the CD4 count remained low. On day 57, blood culture revealed the presence of *Enterococcus faecium* and *Candida parapsilosis. Candida glabrata* was detected in the blood cultures on day 59. Despite continued treatment with antibiotics and antifungals, the patient died of septic shock on day 86 (Fig. S1, Supplemental Digital Content, https://links.lww.com/MD/R542).

## 3. Discussion

NTM prevalence has been increasing globally, with multiple NTMs commonly detected in sputum samples in routine clinical practice. A study by Kim et al observed mixed infections with 3 species of mycobacteria in only 0.99% of cases among patients with pulmonary NTM infections.^[6]^ *Mycobacterium tilburgii* is particularly difficult to identify and has been rarely reported. In our case, the organism was visible in the stained specimens but could not be cultured. Although *M. intracellulare* and *M. kansasii* were isolated from the sputum samples, they were not detected in the blood or bone marrow samples, whereas *M. tilburgii* was confirmed genetically from these specimens. These findings suggest that the disseminated infection was caused predominantly by *M. tilburgii*, whereas the pulmonary lesions may have resulted from concurrent NTM infection. Ultimately, the patient was diagnosed with disseminated *M. tilburgii* and mixed pulmonary NTM infections based on NGS.

*Mycobacterium tilburgii* is an independent species within the *M. simiae* complex and is notably difficult to culture on artificial media. A PubMed search at the time of writing (October 2024) using the keyword “*M. tilburgii*” revealed 16 reported cases.^[3,7]^ *Mycobacterium tilburgii* was detected in the bone marrow in 6 cases, including the current case (Table 4).^[7–11]^ This infection is more prevalent in Europe compared with other regions and is more frequently observed in middle-aged men. Most patients had underlying immunodeficiency, including HIV infection, Mendelian susceptibility to mycobacterial disease, and the presence of anti-interferon-γ autoantibodies.^[3,7]^ These patients also presented with DNTM, with *M. tilburgii* detected in various sites, including the gastrointestinal tract, lymph nodes, and bone marrow. Among these, the patient with *M. tilburgii* detected in the bone marrow exhibited nonspecific findings such as fever and abnormal laboratory findings. Our patient exhibited nonspecific symptoms such as fever and abnormal laboratory findings, consistent with the previously reported features of disseminated *M. tilburgii* infection. However, owing to the patient underlying conditions and complex clinical background, a direct causal relationship could not be established. Detection of *M. tilburgii* in the sputum sample collected on hospital day 30 indicates that *M. tilburgii* infection may also involve the respiratory tract. Although most reported cases describe disseminated disease, our findings suggest the possibility of pulmonary infection, warranting recognition of *M. tilburgii* as a potential respiratory pathogen in severely immunocompromised individuals.

**Table 4:** Case reports of *Mycobacterium tilburgii* isolated from the bone marrow.

Case	References	Age (years)	Sex	Underlying disease	Site of infection	Initial treatment regimen	Drug modification	Outcome
1	Schepers et al^[8]^	33	Female	IL-12Rβ1 deficiency	BM and LN	CLR, EMB, and RIF	CLR, CPX	Survived
2	Temmerman et al^[9]^	41	Male	Sarcoidosis, steroid use	BM, LN, liver, lung, and spleen	AMK, CLR, EMB, LVX, and RIF	CLR, EMB, LVX, LZD, RIF+IFN-γ	Survived
3	Heyckendorf et al^[10]^	41	Male	HIV	BM, LN, and duodenum	AZM, EMB, and RBT	AZM, EMB, RBT+LZD +MFX+AMK+CLR + IL-2 →AZM, EMB, and MFX	Survived
4	Doesschate et al^[11]^	57	Male	Interstitial nephritis, sarcoidosis, and steroid use	BM, LN, BAL, and ascites	CLR, EMB RIF, and OFX	AMK, CLF, and MFX + IFN-γ	Died
5	Yuan et al^[7]^	33	Male	HIV	BM, LN, and duodenum	MFX, CLR, EMB, and RBT	MFX, CLR, EMB, RBT, and AMK	Survived
Our case		40s	Male	HIV	BM, lung, and blood	CLR, EMB, and RBT		Died

AMK = amikacin, AZM = azithromycin, BAL = bronchoalveolar lavage, BM = bone marrow, CLF = clofazimine, CLR = clarithromycin, CPX = ciprofloxacin, EMB = ethambutol, HIV = human immunodeficiency virus, IFN-γ = interferon-γ, IL-12Rβ1 = interleukin-12 receptor β1, IL-2 = interleukin-2, LM = lymph node, LVX = levofloxacin, LZD = linezolid, MFX = moxifloxacin, OFX = ofloxacin, RBT = rifabutin, RIF = rifampicin.

In all reported cases of *M. tilburgii*, acid-fast bacilli were detected in smears but not in cultures on artificial media. Therefore, genetic analyses, including those of 16S rRNA, internal transcribed spacer, *hsp65*, and *rpoB* genes, should be used for identification.^[2,3]^ Only 2 reported cases of *M. tilburgii* were identified using NGS, including the present case.^[7]^ Although the detected mycobacteria showed only 96% similarity with *M. simiae* in the *hsp65* gene, they demonstrated 100% identity with *M. tilburgii* in the 16S rRNA gene. Because 16S rRNA is the most reliable taxonomic marker for *M. tilburgii*, these findings confirmed the organism as *M. tilburgii*, consistent with previous reports describing similar gene divergence within the *M. simiae* complex. Although clarithromycin, ethambutol, rifampicin, rifabutin, and quinolone antibiotics were used,^[3]^ no cases of *M. tilburgii* have documented drug susceptibility data. Therefore, a standardized treatment regimen has not been established. Review of reported cases indicates that all survivors received regimens containing either a fluoroquinolone or an aminoglycoside, whereas our patient did not. This difference may have contributed to treatment failure, underscoring the potential importance of including these agents in the management of suspected *M. tilburgii* infections.

Early diagnosis and effective treatment of disseminated *M. tilburgii* infections are crucial.^[7]^ In our case, clarithromycin, ethambutol, and rifabutin were administered as the standard therapy for suspected disseminated MAC. If the NGS result identifying *M. tilburgii* had become available before the patient death, we would have considered adding moxifloxacin rather than amikacin to the ongoing regimen, based on the regimens followed for previously reported survivors of disseminated *M. tilburgii* infection, most of whom received a fluoroquinolone (typically moxifloxacin) as part of combination therapy.^[7,9]^ *M. tilburgii* is phylogenetically close to *M. genavense* and *M. avium-intracellulare* complex, organisms that are moderately susceptible to fluoroquinolones but less predictably responsive to aminoglycosides. In addition, our patient exhibited severe pancytopenia and a low platelet count (3.2 × 10^4^/µL), making parenteral amikacin potentially hazardous owing to nephrotoxicity and ototoxicity. Therefore, addition of oral moxifloxacin would have been a safer and more rational option than adding amikacin, with careful monitoring for QT prolongation and hepatic toxicity. Even upon earlier identification, treatment options remain limited owing to the absence of standardized regimens or susceptibility data. In our case, sepsis was ultimately considered the direct cause of death, and it remains unclear whether earlier identification of *M. tilburgii* would have markedly improved the prognosis. Nevertheless, we believe that early identification of the pathogen is of great significance. In retrospect, there were several points during the diagnostic process at which *M. tilburgii* infection could have been suspected earlier. In the following section, we thus examine the specific clues and decision-making points in our case that might have facilitated earlier diagnosis. For example, crossbanding-like patterns were observed in the smear microscopy of the sputum sample from this patient; however, PCR analysis identified *M. intracellulare*. The PCR results negated the possibility that *M. intracellulare* caused DNTM infection. *Mycobacterium kansasii* crossbanding has been reported in smear microscopy, suggestive of a mixed pulmonary NTM.^[12]^ In the present case, acid-fast bacilli were detected in the blood and bone marrow smear microscopy. However, crossbanding of *M. kansasii* was not detected in the smear microscopy of the blood and other specimens. These results indicated the presence of acid-fast bacteria other than *M. kansasii* in blood and bone marrow, suggesting that *M. kansasii* was unlikely to be the main cause of the disseminated infection. Despite the high bacterial load, no growth of acid-fast bacilli was observed in blood cultures. The discrepancy between the smear microscopy and culture results is the key to identifying *M. tilburgii*. However, some NTMs have strict growth conditions that lead to discrepancies between smear microscopy and culture results. *Mycobacterium genavense* and *M. haemophilum* have been reported in patients with DNTM who are infected with HIV.^[13,14]^ Therefore, microbiology laboratories should consider generation time, nutritional requirements, and growth temperature ranges when culturing patient samples.

In the present case, *M. tilburgii* and *M. intracellulare* were detected using NGS. However, *M. kansasii* was not detected in the sputum sample collected on hospital day 30 after initiation of treatment. This case highlights the importance of comprehensive genetic analysis using NGS. When discrepancies arise between smear examination and culture results in cases of DNTM infection among individuals who are immunocompromised, bacterial species with strict growth conditions should be considered. Additional testing and genetic analysis should also be performed to facilitate the early identification of bacterial species.

## 4. Conclusion

This case study presents a case of disseminated *M. tilburgii* infection in a patient with AIDS and a mixed pulmonary NTM infection. When acid-fast bacilli are detected in smear microscopy with no growth observed in cultures, considering the possibility of *M. tilburgii* infection and conducting thorough investigations are necessary. Moreover, given that *M. tilburgii* is a non-culturable acid-fast bacterium and its drug susceptibility remains undocumented, further research is needed to enhance understanding and guide effective treatment.

## Acknowledgments

We thank all members of the microbiology laboratory who assisted us in our research.

## Author contributions

**Conceptualization:** Yuki Sakai.

**Data curation:** Kazuo Umezawa.

**Formal analysis:** Yuki Sakai, Miki Miyazawa, Akifumi Hisada, Yukari Tanabe, Akiko Takaki.

**Funding acquisition:** Satomi Asai.

**Investigation:** Yuki Sakai.

**Project administration:** Satoshi Arakawa.

**Resources:** Hidetaka Yanagi.

**Supervision:** Tsukasa Nozaki, Kentaro Wakamatsu, Satomi Asai.

**Visualization:** Kentaro Wakamatsu.

**Writing – original draft:** Yuki Sakai.

**Writing – review & editing:** Miki Miyazawa, Satoshi Mitarai, Kentaro Wakamatsu, Satomi Asai.

## Supplementary Material
