# Supplementary material for: Disseminated Mycobacterium tilburgii infection complicated by pulmonary non-tuberculosis mycobacteriosis in a patient with acquired immunodeficiency syndrome: A case report and literature review
Source: Medicine (Baltimore). 2026 Mar 13;105(11):e47989. doi: 10.1097/MD.0000000000047989 (PMC12991772; doi:10.1097/MD.0000000000047989)
Supplement: Supplementary file 1 [file medi-105-e47989-s001.docx]

**Figure S1.** Clinical Course during Hospitalization

Regarding the clinical course of the patient from admission to death/discharge. The content includes fever, white blood cell count, detected bacteria, and antimicrobial agents.
